# Ciliary G-Protein Coupled Receptor Signaling in Polycystic Kidney Disease

**DOI:** 10.3390/ijms26114971

**Published:** 2025-05-22

**Authors:** Raghad Buqaileh, Lubna A. Alshriem, Wissam AbouAlaiwi

**Affiliations:** 1 Department of Pharmacology and Experimental Therapeutics, University of Toledo, Toledo, OH 43614, USA; raghad.buqaileh@rockets.utoledo.edu (R.B.); lubna.alshriem@rockets.utoledo.edu (L.A.A.); 2 Department of Clinical Pharmacy, Faculty of Pharmacy, Jordan University of Science and Technology, P.O. Box 3030, Irbid 22110, Jordan

**Keywords:** polycystic kidney disease, primary cilia, GPCR, polycystin, ADPKD

## Abstract

Polycystic kidney disease (PKD), a ciliopathy caused primarily by mutations in the *Pkd1* and *Pkd2* genes, disrupts renal structure and function, leading to progressive renal failure. The primary cilium, a sensory organelle essential for cellular signaling, plays a pivotal role in maintaining renal function. Among its signaling components, G-protein-coupled receptors (GPCRs) within the cilium have gained significant attention for their localized functions and their contribution to PKD pathogenesis. Dysfunction of ciliary GPCR signaling alters key downstream pathways, including mammalian target of rapamycin (mTOR), cyclic adenosine monophosphate (cAMP), and calcium homeostasis, exacerbating cyst formation and disease progression. Additionally, interactions between ciliary GPCRs and PKD-associated proteins, such as Polycystin-1 (PC1) and Polycystin-2 (PC2), underline the complexity of PKD mechanisms. Recent advances highlight GPCRs as promising therapeutic targets for ciliopathies, including PKD. Emerging GPCR modulators and drugs in clinical trials show the potential to restore ciliary signaling and attenuate disease progression. This paper explores the physiological functions of ciliary GPCRs, their mechanistic links to PKD, and the therapeutic implications of targeting these receptors, offering insights into future research directions and therapeutic strategies for PKD.

## 1. Introduction

**Figure 1:**
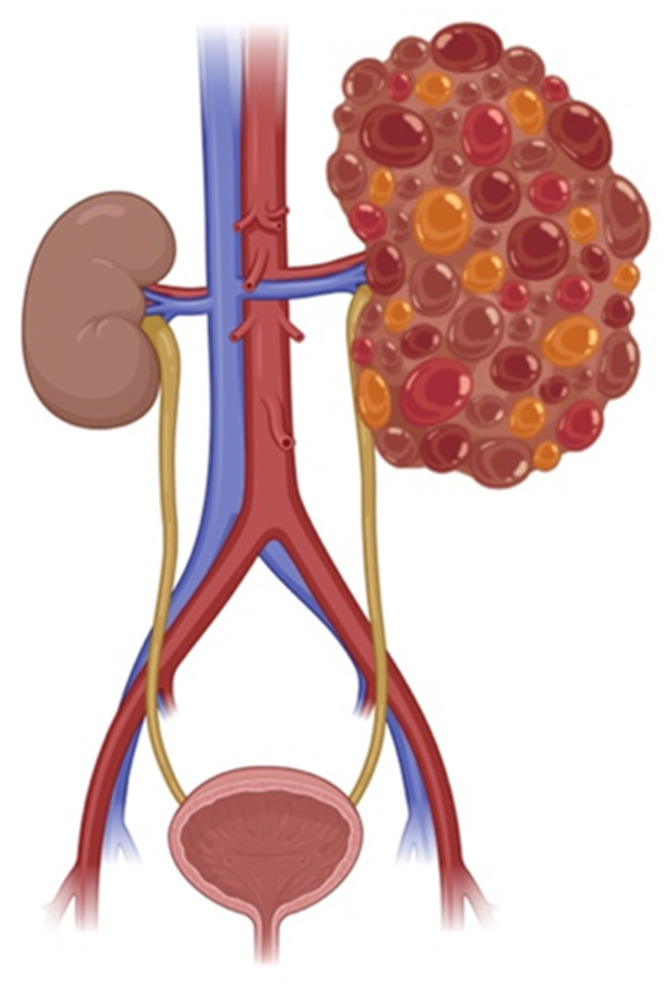
Comparison of a normal kidney and a polycystic kidney. The schematic illustrates the structural differences, highlighting the presence of multiple fluid-filled cysts in the polycystic kidney, which disrupt normal tissue architecture and function. Created with BioRender.com.

Polycystic kidney disease (PKD) is an inherited disorder characterized by the development of multiple fluid-filled cysts that arise from the epithelial cells lining the kidney tubules. Cyst formation typically begins with localized expansion of segments of the renal tubules. These cysts progressively enlarge over time, driven by active fluid secretion into the cyst lumen and proliferation of the cyst-lining epithelial cells. As they grow, cysts expand beyond the dimensions of the original tubules, compressing and displacing surrounding renal tissue. This ongoing disruption of normal kidney architecture ultimately impairs kidney function and can lead to renal failure [1] (Figure 1). PKD also causes extra-renal manifestations, such as cerebral and aortic aneurysms, hepatic and pancreatic cysts, and cardiovascular diseases. These outcomes are primarily linked to mutations in either the Pkd1 or Pkd2 genes, which encode Polycystin-1 (PC1) and Polycystin-2 (PC2), respectively [2]. PC1, a large integral membrane protein with a GPCR-like domain, regulates cell growth, adhesion, and renal tubular development. The membrane topology of PC1 is considered similar to that of GPCRs because it contains an intracellular C-terminus and a long extracellular N-terminus, and it spans the membrane multiple times, although not with the canonical 7-transmembrane domain architecture of classic GPCRs. Mutations in *Pkd1* cause the more severe autosomal dominant PKD type 1 (ADPKD1) [3,4,5]. PC2, a calcium-permeable ion channel, partners with PC1 via its C-terminal and regulates calcium-dependent signaling essential for cellular homeostasis [6,7].

**Figure 2:**
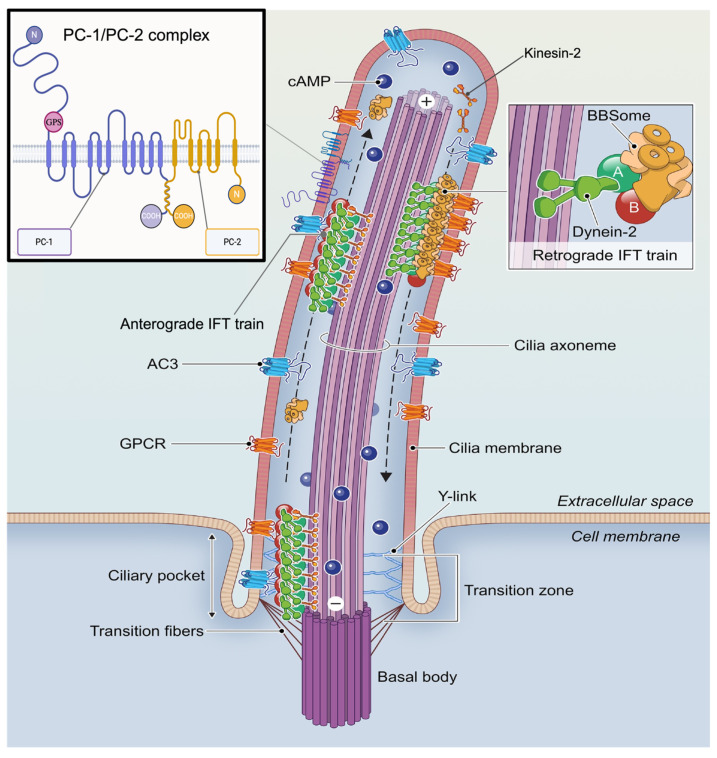
Structural and molecular components of the primary cilium in kidney epithelial cells. This schematic illustrates key features of the cilium, including the ciliary membrane enriched with signaling molecules such as G protein–coupled receptors (GPCRs), adenylate cyclase 3 (AC3), and downstream cAMP signaling pathways along the axoneme. Bidirectional intraflagellar transport (IFT) is shown, with anterograde transport mediated by kinesin-2 and retrograde transport by dynein-2, emphasizing the dynamic trafficking necessary for ciliary maintenance and function. The localization of Polycystin-1 (PC1) and Polycystin-2 (PC2) within the cilium is also depicted. PC1 is shown with large extracellular domains and multiple membrane-spanning regions, while PC2 is illustrated as a typical transmembrane protein. The N-terminal domains of PC1 and PC2 are positioned extracellularly, while their C-terminal (carboxy terminus) domains reside in the cytoplasm. GPS: GPCR Proteolysis Site, N: N-terminus; COOH, Carboxy terminus. modified using biorender.com from [19].

As illustrated in Figure 2, PC1 and PC2 localize to the primary cilium of kidney epithelial cells, forming a mechanosensory complex that detects fluid flow and modulates signaling cascades that control cell proliferation and tubule architecture [8,9]. *Pkd1* mutations typically result in earlier onset and more severe disease than *Pkd2* mutations due to PC1’s upstream role in signaling [8]. Disruption of either polycystin function or primary cilia integrity independently triggers cyst formation, emphasizing their importance in renal homeostasis [10].

Beyond defective ciliary polycystin signaling, PKD pathogenesis involves dysregulation of multiple signaling pathways including mTOR, EGFR, and MAPK, which promote cyst expansion. Metabolic defects such as mitochondrial dysfunction, altered glucose metabolism, and oxidative stress further accelerate disease progression by fostering an environment conducive to abnormal proliferation [7,10]. G-protein coupled receptors (GPCRs), many of which are localized to primary cilia, are emerging as key regulators of these pathogenic pathways. Their role in controlling cell proliferation, fluid secretion, and ciliary function makes them promising therapeutic targets in PKD [11]. Studies have demonstrated that certain GPCRs can be directly activated by reactive oxygen species (ROS) both in vitro and in vivo. This indicates that ROS may not only contribute to cellular stress but also modulate GPCR activity, thereby influencing receptor signaling in both physiological and disease settings [12]. This review aims to explore the emerging roles of ciliary GPCRs in the pathogenesis of polycystic kidney disease (PKD), with a focus on their localization, signaling mechanisms, and therapeutic potential. By integrating recent findings, we seek to clarify how dysfunction in these receptors contributes to PKD progression and highlight promising avenues for future research and drug development.

## 2. Primary Cilia and Their Role in Cellular Signaling

Primary cilia are microtubule-based, non-motile sensory organelles extending from the surface of most vertebrate cells, especially those in the kidney, retina, and embryonic tissues [13,14]. They detect environmental stimuli and convert them into intracellular signals that modulate cellular processes [15]. Structurally, they consist of a basal body and axoneme and range from 2 to 10 μm in length (up to 200 μm in olfactory neurons) [16]. Their membrane contains a distinct set of receptors, ion channels, and signaling proteins that coordinate tissue-specific functions through mechanical or chemical stimulation [17,18]. The spatial organization and trafficking of signaling modules to the cilium underlie its sensory function. Figure 2 illustrates the organization of the primary cilium, including the localization of GPCRs, adenylyl cyclase 3 (AC3), and directional transport systems essential for ciliary signaling [19]. Major signaling pathways localized to cilia include Hedgehog, WNT, GPCR, receptor tyrosine kinase (RTK), transforming growth factor-β (TGF-β), and bone morphogenetic protein (BMP) signaling. For example, olfactory cilia use GPCR-mediated signaling via cAMP to transduce environmental cues into physiological responses [20]. However, the primary cilia’s molecular mechanism of sensory function is still not completely elucidated.

### 2.1. Ciliary GPCR Structure and Function

GPCRs are the largest class of transmembrane receptors in eukaryotes, with over 800 encoded in the human genome [21,22]. These receptors consist of a single polypeptide chain forming seven transmembrane domains, with extracellular N-terminal and intracellular C-terminal ends, and are activated by diverse ligands [21,23,24].

G proteins consist of three subunits: Gα, Gβ, and Gγ. These subunits form a heterotrimeric complex that transmits intracellular signals upon stimulation by G protein-coupled receptor (GPCR) ligand binding. In the resting state, the G protein exists as an inactive heterotrimer (Gα-GDP bound to Gβγ). Upon ligand binding to the receptor, a conformational change in the GPCR activates the Gα subunit, promoting the exchange of GDP for GTP. This activation triggers the dissociation of the heterotrimer into two active components: Gα-GTP and the Gβγ dimer, each of which can independently regulate various downstream effectors such as adenylyl cyclase, cGMP phosphodiesterase, and phospholipase C. The signaling is terminated when the intrinsic GTPase activity of Gα hydrolyzes GTP to GDP, allowing Gα-GDP to reassociate with Gβγ, reforming the inactive heterotrimeric G protein complex [25,26].

GPCRs are grouped into four types based on their Gα subunits: Gs, Gi/o, Gq/11, and G12/13. Gs and Gi regulate cAMP production, while Gq activates the PLC pathway [27].These receptors signal over milliseconds to minutes, with downstream messengers modulating essential physiological functions like metabolism, growth, sensory perception, and homeostasis. Notably, GPCRs are the targets of roughly one-third of all FDA-approved drugs [26,28,29].

#### 2.1.1. Physiological Roles of GPCRs

GPCRs are essential for neurotransmission, cardiac output, immune cell trafficking, hormone release, and energy homeostasis. In the brain, they mediate responses to dopamine, serotonin, GABA, and glutamate, influencing mood, memory, and movement [29,30]. In the heart, adrenergic GPCRs modulate heart rate and contractility [31]. Chemokine GPCRs guide immune cells during inflammation and repair [32]. while endocrine GPCRs regulate hormone secretion and metabolic signals [21,33]. Ciliary GPCRs play critical roles in specialized tissues. For instance, olfactory cilia use GPCRs for smell; retinal GPCRs like rhodopsin enable photoreception; and renal cilia use dopamine and serotonin GPCRs to regulate fluid flow and mechanosensation. Disruption in these receptors leads to ciliopathies like PKD, where failure in mechanotransduction contributes to cyst development [34].

#### 2.1.2. Role of GPCRs in Kidney Function

In the kidney, GPCRs regulate fluid balance, blood pressure, and filtration. Vasopressin receptor 2 (V2R) facilitates water reabsorption in collecting ducts by promoting aquaporin-2 trafficking, a key mechanism in urine concentration [35]. The angiotensin II type 1 receptor (AT1R) governs sodium retention and vasoconstriction as part of the RAAS system [36]. Renal dopamine receptors (D1/D2 types) promote natriuresis by inhibiting sodium transporters, while prostaglandin receptors (EP1/EP2) support renal blood flow and glomerular filtration [37]. GPCRs also mediate renal protection during injury and inflammation [38,39]. Mutations in GPCRs, such as V2R, can lead to nephcrogenic diabetes insipidus (NDI), while dysregulated AT1R or dopamine signaling contributes to hypertension and chronic kidney disease [40,41,42].

### 2.2. Mechanisms Linking Ciliary GPCR Dysfunction to PKD Pathogenesis

Several GPCRs have been identified in primary cilia, including G protein-coupled receptor 161 (GPCR161), Melanocortin 4 receptor (MC4R), Prolactin-releasing peptide receptor (PRLHR), Vasopressin V2 receptor (V2R), Prostaglandin E2 receptor 4 (EP4), Galanin receptor 2 (GALR2), Free fatty acid receptor 4 (FFAR4), and more recently Muscarinic Acetylcholine Receptor 3 (M3R) distributed over the brain, kidney, adipose tissue, and vasculature [43,44]. In the kidneys, primary cilia are found on the apical surface of kidney epithelial cells to serve as fluid flow mechanosensors. However, the mechanism behind their mechanosensory function is still unclear [45]. Studies showed that some GPCRs, including V2R are localized to the basolateral membrane and the primary cilia of renal epithelial cells. Cells of the distal convoluted tubule, collecting duct, distal ascending limb of Henle, macula densa, and connecting tubule all express V2R. Vasopressin activates V2R, initiating Gαs signaling and stimulating adenylyl cyclase 6. This cascade regulates sodium and water reabsorption in the nephron, primarily by controlling the trafficking of aquaporin-2 to the apical membrane of collecting duct principal cells and modulating its gene expression [46]. Additionally, vasopressin activates urea transporters UT-A1 and UT-A3 in the terminal part of the inner medullary collecting duct, enhances epithelial sodium channel activity in the cortical and outer medullary collecting ducts, and increases the expression and activity of the sodium-potassium-chloride cotransporter in the thick ascending limb of Henle [47,48,49]. Furthermore, GPCRs, such as EP1–EP4, are activated by prostaglandin E2. Among these, EP2 and EP4 activate protein kinase A signaling and increase intracellular cAMP. Jin, Daqing et al. demonstrated that EP4, localized to the cilium, activates the cAMP-mediated signaling cascade, which is essential for cilium formation and elongation [50].

Dopamine receptor 5 and somatostatin receptors are other examples of receptors and their associated signaling cascades localized to the primary cilia. Dopamine receptor 5 (DR5) is a G-coupled protein receptor [51]. Upadhyay et al. demonstrated that DR5 localized to primary cilia in renal proximal epithelial cells. Activation of DR5 by fenoldopam, a selective DR5 dopamine agonist, resulted in a significant increase in primary cilia length. Thus, it has been suggested to promote the ciliary response to mechanical stimulation by fluid flow [52]. However, the effect of cilia-independent dopamine receptors is different. Dopamine receptors are responsible for the nuclear export of histone deacetylase 5 (HDAC5), which helps maintain the renal epithelial architecture by suppressing the MEF2C target genes. Parama P. et al. found that dopamine receptor antagonists stimulate the export of HDAC5, resulting in significantly reduced cystic growth and cell proliferation in PKD mice [53].

Somatostatin receptors (SSTR) are a family of five GPCRs distributed in the lung and kidney tissue (SSTR1-5). Somatostatin (SST) is a hormone that binds to SSTR to activate the inhibitory G-protein (Gi) and modulate several secondary signaling [54]. These receptors are predominantly distributed in the tubular epithelium and glomerular mesangial cells. The SSTR1 and SSTR2 are widely expressed throughout the distal nephron and collecting duct in normal human kidney [55]. Meanwhile, SSTR3, 4, and 5 are expressed in the proximal tubules [56]. The role of SST and its secondary signaling in the kidney’s primary cilia is not sufficiently addressed in the literature. One study showed that somatostatin decreases the cAMP formation by antagonizing the vasopressin effect in cortical, medullary collecting tubules, and in the medullary thick ascending limb of Henle’s loop micro-dissected from rat kidney [57].

PC1 has demonstrated the ability to sense physical stimuli from the surrounding environment and translate them into a biochemical signaling output inside the cell. PC1 is preferentially bound to several classes of Gα subunits at a specific site called the G protein-binding domain (GBD), which is a unique 20-amino acid region at the COOH terminus [58]. Furthermore, PC1 possesses a GPCR proteolysis site (GPS), a highly conserved autoproteolytic site, within a GPCR-autoproteolytic inducing (GAIN) domain on the first transmembrane span’s NH2-terminal side. The GAIN domain was found in cell adhesion GPCRs to maintain tubular epithelial cell morphology [59]. The COOH terminus of PC1 is known to mediate the interaction of PC1 and PC2, which is involved in the regulation of ion transport and calcium signaling [60]. However, the exact molecular mechanism underlies PC1’s activity in renal epithelial cells’ primary cilia is still unknown.

**Figure 3:**
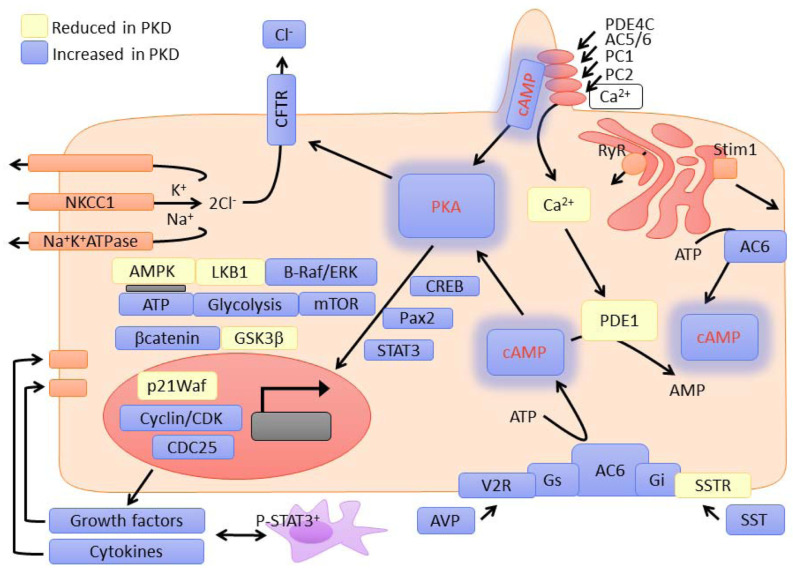
Overview of dysregulated signaling pathways in ADPKD. Activation of vasopressin V2 receptors (V2R) and inhibition of somatostatin receptors (SSTR) influence adenylyl cyclase (AC6) activity and intracellular cAMP levels. Elevated cAMP activates protein kinase A (PKA), driving downstream signaling via CREB, mTOR, STAT3, and Pax2, and promoting cystogenesis. Calcium flux through PC1/PC2 and PDE1-mediated degradation of cAMP are impaired. Blue = upregulated in PKD; yellow = downregulated. Used with a permission from [11].

Defective GPCR signaling in cilia disrupts essential cellular processes such as cell proliferation, fluid regulation, and mechanosensory functions, which are crucial for maintaining renal epithelial homeostasis [61]. One critical pathway affected by GPCR dysfunction is the cAMP signaling pathway. Aberrant GPCR activity in primary cilia has been linked to elevated cAMP levels in PKD. This increase in cAMP promotes aberrant cell proliferation and fluid secretion into cysts through activation of protein kinase A (PKA) and downstream effectors, such as the cystic fibrosis transmembrane conductance regulator (CFTR) channel [62]. These disruptions amplify cyst expansion and contribute to kidney enlargement in PKD Figure 3 provides a visual summary of major signaling alterations in PKD related to ciliary GPCR dysfunction, highlighting the central role of the cAMP-PKA pathway and its interactions with calcium, mTOR, and metabolic regulators [11].

GPCR dysfunction also impairs calcium signaling, which is tightly regulated by ciliary GPCRs. Calcium homeostasis in primary cilia plays a pivotal role in regulating cellular processes such as apoptosis, proliferation, and polarity. Dysregulated calcium signaling in PKD can suppress calcium-sensitive phosphodiesterases, leading to an imbalance in cAMP levels and exacerbating cystogenesis [63]. Furthermore, the interaction of ciliary GPCRs with the mTOR pathway highlights another mechanism by which GPCR dysfunction drives PKD pathogenesis. Abnormal GPCR signaling can hyperactivate mTOR, a key regulator of cell growth and metabolism, resulting in enhanced proliferation and cyst expansion. Notably, the mTOR inhibitor rapamycin has been shown to reduce cyst growth in preclinical PKD models, underscoring the significance of this pathway [64].

The relationship between ciliary GPCRs and PKD-associated proteins, such as PC1 and PC2, plays a central role in cyst formation and kidney dysfunction. PC1, an atypical GPCR, and PC2, a TRP channel, form a functional complex within primary cilia, where they regulate calcium influx and mechanosensory signaling. Disruption of this complex due to mutations in the *Pkd1* or *Pkd2* genes impairs calcium signaling, creating a permissive environment for cyst growth [9].

Ciliary GPCRs influence PC1 and PC2 activity through their interactions with G-proteins and secondary signaling pathways. For instance, GPCRs localized in cilia can modulate intracellular calcium levels via PC2 channels, maintaining the balance between cell proliferation and apoptosis. When this regulation fails, cells adopt a hyperproliferative state, contributing to cyst formation and expansion [65,66]. Moreover, PC1 has been shown to inhibit mTOR signaling through interactions with tuberin (TSC2), a key component of the mTOR pathway. Dysfunctional GPCR signaling can disrupt this interaction, leading to unchecked mTOR activity and accelerated cyst growth [67].

Emerging evidence suggests that ciliary GPCR signaling may also influence the trafficking and localization of PC1 and PC2 within primary cilia. The mis-localization of these proteins, caused by defective GPCR signaling, can impair the sensory and regulatory functions of primary cilia, further exacerbating PKD pathology. Understanding these intricate interactions provides valuable insights into the molecular mechanisms driving PKD and highlights potential therapeutic targets for mitigating disease progression [44].

### 2.3. Mechanosensation and Fluid Flow Signaling in Ciliary GPCRs and PC1

PC1 is uniquely positioned to act as a mechanosensor at the ciliary membrane, enabling renal epithelial cells to transduce luminal shear stress into intracellular signals. Structurally, PC1 contains GPS site and a GAIN domain, reminiscent of adhesion GPCRs, but its mechanosensitivity appears independent of canonical ligand binding. Ciliary deflection caused by fluid flow is proposed to induce conformational changes in the extracellular region of PC1, which are transmitted to its intracellular C-terminal tail. This tail interacts with Gα subunits and cytoplasmic signaling complexes, allowing PC1 to initiate intracellular responses to mechanical stimuli without chemical ligands [61,68].

Upon flow-induced activation, PC1 modulates intracellular signaling in part through its interaction with PC2, promoting calcium influx. However, emerging evidence suggests that PC1 may also act independently to activate G proteins directly, with the G protein-binding domain at its cytoplasmic terminus required for normal mechanotransduction [3]. This mechanism allows PC1 to regulate downstream effectors such as adenylyl cyclase, mTOR, and planar cell polarity regulators in a flow-dependent manner. Notably, loss of PC1 does not simply eliminate calcium influx, it abolishes the ability of cells to interpret flow, underscoring its role as a true biomechanical sensor, rather than a passive cofactor [69].

Recent studies suggest that certain ciliary GPCRs may, themselves, be sensitive to mechanical stimuli, either through conformational activation induced by membrane stretch or via flow-induced changes in ciliary lipid microdomains. GPR68 and GPR161, for instance, exhibit flow-responsive behavior in other epithelial systems and may be similarly activated in renal cilia [70]. These receptors could function in parallel to or downstream of PC1, amplifying or modulating mechanosensory outputs. However, direct evidence linking these GPCRs to cyst initiation via mechanotransduction in PKD remains limited.

Loss of PC1’s mechanosensory function has been directly linked to early cyst initiation. In normal epithelia, flow-induced PC1 activation constrains cell proliferation and maintains planar polarity through coordinated calcium and G protein signaling. In *Pkd1*-deficient cells, this mechanical feedback is lost, rendering cells unresponsive to physiological shear stress. As a result, intracellular cAMP accumulates unchecked, promoting PKA-mediated proliferation and fluid secretion. Concurrently, impaired calcium influx destabilizes cytoskeletal architecture and disrupts oriented cell division, both of which contribute to abnormal tubule dilation and cyst formation. These mechanistic disruptions occur prior to overt cyst development, underscoring that defective flow sensing via PC1 is not merely a consequence of cyst growth, but an initiating factor in ADPKD pathogenesis [9,71].

Collectively, these findings indicate that mechanosensation in the primary cilium is not confined to PC1-PC2 function but may involve a broader network of mechanically responsive receptors. Understanding the flow-dependent behavior of these GPCRs, and how they integrate or compete with PC1 signaling, may provide critical insight into the earliest steps of cystogenesis and reveal novel therapeutic targets upstream of current cAMP-directed interventions.

## 3. Therapeutic Implications and Drug Development

In PKD, clinical features such as hypertension and proteinuria are strongly linked to underlying molecular mechanisms. Hypertension is one of the earliest and most common manifestations, often resulting from activation of the RAAS elevated vasopressin levels and sodium retention are influenced by dysregulated GPCR signaling, particularly through V2R and AT1R pathways [41,42]. Proteinuria, another hallmark of disease progression, arises from glomerular and tubular damage associated with increased intracellular cAMP, calcium signaling disturbances, and oxidative stress [72]. These features not only reflect the pathophysiological burden of PKD but also serve as critical markers for evaluating disease severity and therapeutic response.

Although several GPCRs have been successfully targeted to treat many human diseases, limited phenotypes have been considered for the treatment of PKD. The vasopressin V2 and the somatostatin receptors are the two majors targeted GPCRs for the treatment of PKD. Vasopressin binds and activates three different vasopressin receptors V1a, and V1b, which are coupled to Gαq/11 proteins, and V2 receptors, which are coupled to Gαs proteins [47]. The effects following V2R activation are mediated by cAMP, whilst the effects following V1aR and V1bR activation are mediated by calcium signals. V2 receptors are highly distributed in the kidneys and are mainly expressed in the thick ascending limb of the loop of Henle, macula densa, distal convoluted tubule, connecting tubule, and collecting duct. The V1a receptors are found in vascular smooth muscle cells of interlobular arteries and efferent arterioles, interstitial cells of the medulla, descending vasa recta, and collecting duct principal and alpha intercalated cells [49]. The balanced interaction between the effect of V2R and V1a is critical for maintaining kidney function. Activation of V2R enhances water and sodium reabsorption, which contributes jointly to urine concentration. V1aR counteracts the V2R effects by increasing the production of prostaglandins by collecting duct cells that indirectly attenuate the adenylate cyclase response to V2R stimulation, leading to attenuating the antidiuretic and anti-natriuretic effects of V2R [48]. High levels of vasopressin are found in the early stages of PKD due to impaired urine concentrating capacity and increases further in late PKD, where the vasopressin accumulates in renal insufficiency [73,74]. Vasopressin is hypothesized to be involved in the pathophysiology of kidney cyst development in ADPKD. It was found that increasing the cAMP production in the principal cells following V2R activation stimulates cyst production by promoting fluid secretion and activation and proliferation of cyst-derived cells [75]. Boertien et al. studied the association between the plasma level of Copeptin, which is a surrogate marker for vasopressin, at baseline, and the prognosis of kidney function during the follow-up period in patients with ADPKD. They showed that in ADPKD patients, the plasma concentration of Copeptin is associated with a decline in kidney function, assessed as either a change in inulin clearance (mGFR) during short-term follow-up or as a change in Modification of Diet in Renal Disease (MDRD) equation estimated GFR (eGFR) during long-term follow-up [76]. Many studies have addressed the effectiveness of V2R antagonists in treating and decreasing the prognosis of cyst formation in PKD [77,78]. The effect of different dosages of the V2R antagonist in a PKD1 knockout mouse model for ADPKD at different stages of the disease was investigated and the results showed that initiation of V2R antagonist at the advanced stage of ADPKD lacked reno-protective effects and had less pronounced physiologic effects than early initiation. After 3 weeks of high-dose treatment, the cyst ratio and kidney weight were significantly decreased compared to the control group. However, after 6 weeks of treatment, these results were not significant anymore, even at a high dose. Thus, this study suggests that intervention with a V2R antagonist should be initiated early in ADPKD and that it might be necessary to increase the dosage of this drug further later in the disease to reduce cyst growth [77]. Tolvaptan is an FDA-approved V2R antagonist for the treatment of PKD, which reduces intracellular cAMP levels in the kidney’s collecting duct and has been demonstrated to successfully slow down the progression of ADPKD. A study was conducted to investigate the effect of graded concentrations of Tolvaptan on intracellular cAMP, cell proliferation, and transcellular chloride secretion using human ADPKD cyst epithelial cells. The results showed that Tolvaptan caused a concentration-dependent inhibition of vasopressin-induced cAMP production with an apparent half-maximal inhibitory concentration IC_50_ of ~10^−10^ M indicating high potency. In addition, Tolvaptan inhibited the vasopressin-induced cell proliferation and chloride secretion and decreased in vitro cyst growth of ADPKD human cells [79]. Several clinical trials have confirmed the efficacy of Tolvaptan for the treatment of ADPKD. A phase 3, multicenter, double-blind, placebo-controlled, 3-year trial was conducted for 1445 ADPKD patients that were randomly divided into Tolvaptan-treated and placebo groups, aimed to measure the annual rate of change in the total kidney volume and the rate of kidney function decline. The results showed that the increase in total kidney volume in the Tolvaptan-treated group was 2.8% per year which is significantly lower than the placebo group where the increase in kidney volume was 5.5% per year. Furthermore, Tolvaptan was associated with a slower decline in kidney function compared to placebo, which was evaluated by measuring the serum creatinine. However, Tolvaptan was associated with a higher discontinuation rate, owing to adverse events [80]. An extension phase 3 clinical trial was conducted to evaluate the efficacy and safety of Tolvaptan in patients with later-stage ADPKD. The efficacy was evaluated by measuring the glomerular filtration rate (GFR), and the safety was evaluated by measuring the alanine aminotransferase and bilirubin levels that represent hepatotoxicity. The results showed that the change from baseline in the estimated GFR was −2.34 mL per minute per 1.73 m^2^ in the Tolvaptan-treated group, as compared with −3.61 mL per minute per 1.73 m^2^ in the placebo group (*p* < 0.001). A significant elevation of alanine aminotransferase level was found in 5.6% of patients in the Tolvaptan-treated group, while 1.2% of patients had this elevation in the placebo group. No significant elevation in bilirubin levels was found in both groups. This study showed that Tolvaptan slowed the progressive loss of renal function in patients with ADPKD at later-stage ADPKD [81]. Although numerous preclinical research and clinical trials supported the strong case for using V2R antagonists in PKD, there is little evidence to support inhibiting V1a receptors. Activation of V1a receptors coupled to Gαq/11 proteins enhances calcium signaling and prostaglandin synthesis and, based on the prostaglandin receptor-activated and its downstream mechanisms, results in either detrimental or beneficial effects on PKD. However, many studies support that the V1a antagonists have a reno-protective effect in chronic kidney disease. The effect of a dual V1a and V2 receptor antagonist was assessed on renal disease progression initiated three weeks after nephrectomy in Sprague Dawley rats. The results showed that V1a and V2 receptor antagonists have a renal-protective effect by lowering blood pressure and proteinuria and partial recovery of glomerular and tubular alterations and renal functional impairment [82]. Since the effect of the V2R antagonist is limited to renal tubular cells in the distal nephron and collecting duct, which expresses the V2R, the cysts that originate from other nephron segments will probably not be affected. Further studies are needed to address the specific role of V1a receptor antagonists in PKD.

Somatostatin binds to and activates five different GPCRs, SSTR1-5. The effects following SSTR1-5 activation are mediated by cAMP. Many studies support that somatostatin may inhibit the growth of cysts by acting on several levels, including inhibiting cAMP generation and antagonizing vasopressin-induced cAMP generation and water permeability in collecting ducts [83,84]. Furthermore, it inhibits the secretin-induced cAMP generation and fluid secretion in cholangiocytes and suppresses the vascular endothelial growth factor, and other cytogenic growth factors causing downstream signaling of their receptors in the liver [85,86]. Octreotide, lanreotide, and pasireotide are somatostatin analogs with long half-lives that have been developed for clinical use. Octreotide has been found to activate SSRT2, 3, and 5, decrease the cAMP levels and reduce cyst growth in vitro. In vivo, octreotide lowered cAMP levels in cholangiocytes and serum and decreased the hepatic disease progression. The results showed reductions in liver weight, cyst volume, hepatic fibrosis, and mitotic indices. Comparable effects were shown in the kidneys of PKD rats [87]. Beneficial effects were also found with pasireotide in reducing hepatorenal cystogenesis in rodent models, suggesting its effectiveness in the treatment of polycystic liver and kidney diseases [88]. The effectiveness of Tolvaptan and pasireotide combination therapy was evaluated in the treatment of PKD. The results demonstrated that combination treatment significantly reduced cystic and fibrotic volume and decreased cAMP to normal control levels [89]. Clinical trials of octreotide in ADPKD patients at high risk of end-stage renal disease showed slowed kidney growth and delayed kidney failure progression [90,91]. Systematic review and meta-analysis were performed for clinical trials to evaluate the efficacy of somatostatin analogs treatment in ADPKD and polycystic liver disease. The results showed that the somatostatin analog treatment significantly reduced total liver volume. However, no significant effect was shown in total kidney volume, eGFR, and the progression to end-stage renal failure [92].

Since patients with PKD show abnormal sensory cilia function, which is also related to the cilia structure and length, Kathem et al. studied a targeted therapy focused on primary cilia, dopamine agonists. They conducted both in vivo and in vitro studies to evaluate the effect of fenoldopam (DR5 agonist) on cilia length and function. They found that the activation of primary cilia with fenoldopam induced nitric oxide biosynthesis and increased cilia length within 16 h in a dose-dependent manner. A dose of 10 μmol/L fenoldopam significantly increased cilia length from 2.4 ± 0.1 μm to 3.6 ± 0.2 μm and from 1.3 ± 0.1 μm to 1.6 ± 0.1 μm in wildtype and *pkd1* endothelial cells, respectively. Furthermore, the results showed that dopaminergic activation can improve primary cilia’s mechanosensation function by increasing nitric oxide production in response to fluid shear stress. They also conducted a crossover, multicenter, double-blind, and placebo-controlled clinical study to evaluate the effects of levodopa (a non-specific dopamine agonist) in patients with borderline hypertension and PKD patients with borderline hypertension. The results further indicated that cilia-targeting therapy showed an overall reduction in mean arterial pressure in PKD patients [93]. Table 1 presents a summary of major pre-clinical studies and clinical trial outcomes in managing PKD.

## 4. Current Challenges and Future Directions

Due to the wide range of cellular processes that primary cilia regulate and the large number of GPCRs localized on the cilia with their secondary signaling pathway, there are multiple pathogenic drivers of disease. In some respects, this is beneficial since it provides a wealth of information regarding possible therapeutic targets. However, it becomes difficult to alter one pathway without adversely influencing another because of the crosstalk across pathways and feedback loops. Furthermore, isolating ciliary-specific signaling events is challenging because ciliary GPCRs frequently share signaling pathways with non-ciliary receptors. Developing cilia-specific assays that can distinguish between these pathways is a challenge. Future directions in ciliary GPCR signaling and PKD involve more understanding and identifying the molecular mechanism involved in the pathology of PKD. In addition, the development of targeted drugs that specifically affect ciliary GPCRs may help in slowing the progression and treatment of PKD.

## 5. Conclusions

Ciliary GPCRs are essential regulators of renal physiology, and their dysfunction significantly contributes to the pathogenesis of PKD. Disruption of ciliary GPCR signaling perturbs critical pathways, driving cyst formation and accelerating disease progression. The interaction between GPCRs and PKD-associated proteins, including PC1 and PC2, highlights the intricate molecular mechanisms underlying PKD. While several GPCRs have been implicated in PKD pathophysiology, a vast number of GPCRs remain uncharacterized in the context of PKD and ciliary function. Many of these receptors may localize to the primary cilium and influence disease-related signaling pathways, representing untapped therapeutic potential. Investigating these untested GPCRs holds promise for uncovering novel targets and advancing therapeutic strategies. Despite existing challenges, continued exploration of ciliary GPCRs could lead to significant breakthroughs in PKD treatment, offering new hope for patients affected by this complex disease.

## Figures and Tables

**Table 1:** Summary of major pre-clinical studies and clinical trials outcomes in managing PKD.

Drug	Target and MOA	Pre-Clinical Outcome	Clinical Trial Outcome	References
OPC 31260	V2R antagonist	↓ Cyst ratio and kidney weight after 3 weeks high-dose		[78]
Tolvaptan	V2R antagonist	↓ cAMP, ↓ proliferation, ↓ chloride secretion, ↓ cyst growth	↓ Kidney volume growth (2.8% vs. 5.5% placebo), ↓ function decline	[80,81,82]
RWJ-676070	V1a and V2 receptor antagonists	↓ BP and proteinuria, partial glomerular/tubular restoration	↓ eGFR decline (*p* < 0.001)	[83]
Octreotide	Somatostatin; SSRT2, 3, and 5 agonists	↓ cAMP, ↓ liver/kidney cyst volume, ↓ mitotic index	↓ Kidney growth and function loss in high-risk ADPKD patients	[88,91,92]
Pasireotide	Somatostatin; SSRT2, 3, and 5 agonists	↓ Hepatorenal cystogenesis in rodent models	-	[89]
Tolvaptan and pasireotide -combination	V2R antagonist And SSRT2, 3, and 5 agonists	↓ Cystic and fibrotic volume, normalized cAMP	-	[90]
Fenoldopam	Dopamine- DR5 agonist	↑ Cilia function via NO production in response to shear	-	[93]
Levodopa	Non-specific dopamine agonist	-	↓ BP in PKD patients with borderline hypertension	[93]

PKD: Polycystic kidney disease, MOA: Mechanism of action, V2R: Vasopressin 2 receptor, V1a: Vasopressin 1a receptor, DR5: Dopamine receptor 5, SSRT: Somatostatin receptor. ↑ indicates increase; ↓ indicates decrease.

## Data Availability

Not applicable.

## Abbreviations

The following abbreviations are used in this manuscript:

ADPKD	Autosomal Dominant Polycystic Kidney Disease
ADPKD1	Autosomal Dominant Polycystic Kidney Disease Type 1
ADPKD2	Autosomal Dominant Polycystic Kidney Disease Type 2
AT1R	Angiotensin II Type 1 Receptor
BMP	Bone Morphogenetic Protein
cAMP	Cyclic Adenosine Monophosphate
CFTR	Cystic Fibrosis Transmembrane Conductance Regulator
cGMP	Cyclic Guanosine Monophosphate
CKD	chronic kidney disease
COOH	Carboxy Terminus
DR5	Dopamine Receptor 5
eGFR	Estimated Glomerular Filtration Rate
EP1–EP4	Prostaglandin E2 Receptors 1 to 4
EP4	Prostaglandin E2 receptor 4
FDA	Food and Drug Administration
FFAR4	Free fatty acid receptor 4
GAIN	GPCR-Autoproteolytic Inducing Domain
GALR2	Galanin receptor 2
GBD	G protein-binding domain
GFR	Glomerular Filtration Rate
GPCR	G-Protein Coupled Receptor
GPS	GPCR Proteolysis Site
Gs, Gi, Gq, G12	G Protein Classes
Gα, Gβ, Gγ	G Protein Subunits Alpha, Beta, Gamma
HDAC5	Histone Deacetylase 5
IC50	Half Maximal Inhibitory Concentration
MDRD	Modification of Diet in Renal Disease
mGFR	measured Glomerular Filtration Rate
mTOR	Mammalian Target of Rapamycin
NDI	Nephrogenic Diabetes Insipidus
PC1	Polycystin-1
PC2	Polycystin-2
PKA	Protein Kinase A
PKD	Polycystic Kidney Disease
Pkd1	Polycystic Kidney Disease gene 1.
Pkd2	Polycystic Kidney Disease gene 2.
PLC	Phospholipase C
RAAS	Renin–Angiotensin–Aldosterone system
RTK	Receptor Tyrosine Kinase
SSRT2, SSRT3, SSRT5	Somatostatin Receptor Subtypes 2, 3, and 5
SST	Somatostatin
TGF-β	Transforming Growth Factor Beta
TSC2	Tuberin (Tuberous Sclerosis Complex 2)
UT-A1	Urea Transporter A1
UT-A3	Urea Transporter A3
V2R	Vasopressin V2 Receptor
WNT	Wingless-related integration site
μm	Micrometer
